# NBLDA: negative binomial linear discriminant analysis for RNA-Seq data

**DOI:** 10.1186/s12859-016-1208-1

**Published:** 2016-09-13

**Authors:** Kai Dong, Hongyu Zhao, Tiejun Tong, Xiang Wan

**Affiliations:** 1 Department of Mathematics, Hong Kong Baptist University, Kowloon Tong, Hong Kong; 2 Department of Biostatistics, Yale University, New Haven, 06510 CT USA; 3 Department of Computer Science and Institute of Computational and Theoretical Studies, Hong Kong Baptist University, Kowloon Tong, Hong Kong

**Keywords:** RNA-Seq, Negative binomial distribution, Linear discriminant analysis

## Abstract

**Background:**

RNA-sequencing (RNA-Seq) has become a powerful technology to characterize gene expression profiles because it is more accurate and comprehensive than microarrays. Although statistical methods that have been developed for microarray data can be applied to RNA-Seq data, they are not ideal due to the discrete nature of RNA-Seq data. The Poisson distribution and negative binomial distribution are commonly used to model count data. Recently, Witten (Annals Appl Stat 5:2493–2518, 2011) proposed a Poisson linear discriminant analysis for RNA-Seq data. The Poisson assumption may not be as appropriate as the negative binomial distribution when biological replicates are available and in the presence of overdispersion (i.e., when the variance is larger than or equal to the mean). However, it is more complicated to model negative binomial variables because they involve a dispersion parameter that needs to be estimated.

**Results:**

In this paper, we propose a negative binomial linear discriminant analysis for RNA-Seq data. By Bayes’ rule, we construct the classifier by fitting a negative binomial model, and propose some plug-in rules to estimate the unknown parameters in the classifier. The relationship between the negative binomial classifier and the Poisson classifier is explored, with a numerical investigation of the impact of dispersion on the discriminant score. Simulation results show the superiority of our proposed method. We also analyze two real RNA-Seq data sets to demonstrate the advantages of our method in real-world applications.

**Conclusions:**

We have developed a new classifier using the negative binomial model for RNA-seq data classification. Our simulation results show that our proposed classifier has a better performance than existing works. The proposed classifier can serve as an effective tool for classifying RNA-seq data. Based on the comparison results, we have provided some guidelines for scientists to decide which method should be used in the discriminant analysis of RNA-Seq data. R code is available at http://www.comp.hkbu.edu.hk/~xwan/NBLDA.Ror https://github.com/yangchadam/NBLDA

## Background

RNA-sequencing (RNA-Seq) is a revolutionary technology that uses the capabilities of next-generation sequencing to infer gene expression levels [1–3]. Compared to microarray technology, RNA-Seq has many advantages including the detection of novel transcripts, low background signal, and the increased specificity and sensitivity. Due to reduced sequencing cost, RNA-Seq has been widely used in biomedical research in recent years [4]. In general, three major steps are involved in RNA-seq: (1) RNA is isolated from biopsy or serum sample and segmented to an average length of 200 nucleotides; (2) The RNA segments are converted into cDNA; and (3) The cDNA is sequenced. RNA-seq usually produces millions of short reads, between 25 and 300 base-pairs in length. The reads are then mapped to genomic or transcriptomic regions of interest.

RNA-seq is different to the microarray technology that measures the level of gene expression on a continuous scale. It counts the number of reads that are mapped to one gene and measures the level of gene expression with nonnegative integers. As a result, popular tools that assume a Gaussian distribution in microrray data analysis, such as linear discriminant analysis, may not perform as well as those methods that adopt appropriate discrete distributions for RNA-Seq data. Law et al. [5] recently proposed applying normal-based microarray-like statistical methods to the data transformed from RNA-seq read counts. Simulation studies show that this approach performs as well or better than count-based RNA-seq methods particularly when the number of replicates is large. However, conducting transformation may remove the count nature of the data [6, 7]. McCarthy et al. [8] pointed out that the transformation is not fully tuned to the characteristics of read count data, and provided more detailed reasons why the transformation is inappropriate. First, they stated that for very small counts, they are far from normally distributed after transformation. Second, the strong mean-variance relationship which the count data shows is ignored and this will lead to inefficient inference.

For RNA-Seq data, the Poisson distribution and negative binomial distribution are two common distributions considered in the expression detection and classification. Many methods have been proposed to detect differentially expressed genes, including edgeR [9, 10], DESeq2 [11], baySeq [12], BBSeq [13], SAMseq [14], DSS [15], AMAP [16], sSeq [17], and LFCseq [18]. However, there is less progress in classification using RNA-Seq data until recently. Witten [19] proposed a Poisson linear discriminant analysis (PLDA) which assumes that RNA-Seq data follow the Poisson distribution. Tan et al. [20] further discussed many methods, such as logistic regression and partial least squares, and showed that PLDA is a comparable method. The Poisson distribution is suitable for modeling RNA-Seq data when biological replicates are not available. However, if biological replicates are available, the Poisson distribution may not be a proper choice owing to the overdispersion issue, where the variances of such data are likely to exceed their means [11, 16]. The overdispersion issue can have a significant effect on classification accuracies. In real-world applications, biological replicates can provide more convincing results than technical replicates. Therefore, it is necessary to look for some solutions to take the overdispersion issue into consideration.

We note that Witten [19] has considered this problem and pointed out that the classification accuracy can be further improved for overdispersed data by extending the Poisson model to the negative binomial model. However, to construct an appropriate negative binomial classifier for practical use, two major issues remain to be solved. The first issue is that the probability density function (pdf) of the negative binomial distribution is more complicated than that of the Poisson distribution, which gives rise to a more complicated classifier. The second issue is that the negative binomial distribution contains a dispersion parameter, which controls how much its variance exceeds its mean. To construct the classifier using the negative binomial model, we need to estimate the dispersion parameter. To avoid fitting the complicated negative binomial model, Witten [19] proposed a transformation method for the overdispersed data and found that this method works well if the overdispersion is mild.

In light of the importance of the dispersion in modelling RNA-Seq data with the negative binomial distribution, some dispersion estimation methods have been proposed recently in the literature. For example, Wu et al. [15] proposed a dispersion estimator using the empirical Bayes method and applied it to find differentially expressed genes. Yu et al. [17] proposed a shrinkage estimator of dispersion which shrinks the estimates obtained by the method of moments towards a target value, and also applied it to detect differentially expressed genes. These new methods for estimating the dispersion parameter make it possible to construct a negative binomial classifier to achieve better classification accuracy on RNA-Seq data.

In this paper, we propose a negative binomial linear discriminant analysis (NBLDA) for RNA-Seq data. The main contributions of this paper are in, but not limited to, the following two aspects:

1. We extend Witten’s method [19] by building a new classifier based on the negative binomial model. Under the assumption of independent genes, we define the discriminant score by Bayes’ rule and propose some plug-in rules to estimate the unknown parameters in the classifier.
2. We further explore the relationship between NBLDA and PLDA. A numerical comparison is conducted to explore how the dispersion changes the discriminant score. The comparison results will provide some guidelines for scientists to decide which method should be used in the discriminant analysis of RNA-Seq data.

To demonstrate the performance of our proposed method, we conduct several simulation studies under different numbers of genes, sample sizes, and proportions of differentially expressed genes. Simulation results show that the proposed NBLDA outperforms existing methods in many settings. Three real RNA-Seq data sets are also analyzed to demonstrate the advantages of NBLDA. Specifically, we propose the negative binomial classifier, explore the relationship between NBLDA and PLDA, and present the parameter estimation in Section “Methods”. Simulation studies and real data analysis are conducted in Sections “Results” and “Discussion”, respectively. We conclude the paper with some discussions in Section “Conclusions”.

## Methods

Let *X*_*ig*_ denote the number of reads mapped to gene *g* in sample *i*, *i*=1,…,*n* and *g*=1,…,*G*. To identify which class a new observation belongs to, Witten [19] proposed a PLDA for classifying RNA-Seq data. In this section, we propose a new discriminant analysis for RNA-Seq data by assuming that the data follow the negative binomial distribution.

### Negative binomial linear discriminant analysis

Consider the following negative binomial distribution for RNA-Seq data:

1 $$\begin{array}{@{}rcl@{}} X_{ig} ~\sim~ \text{NB}(\mu_{ig},\phi_{g}),~~~~~\mu_{ig}=s_{i} \lambda_{g}, \end{array} $$

where *s*_*i*_ is the size factor which is used to scale gene counts for the *i*th sample due to different sequencing depth, *λ*_*g*_ is the total number of reads per gene, and *ϕ*_*g*_≥0 is the dispersion parameter. We have *E*(*X*_*ig*_)=*μ*_*ig*_ and $\text {Var}(X_{ig})=\mu _{ig}+\mu _{ig}^{2}\phi _{g}$. Note that the variance is larger than the mean for the negative binomial distribution. Noting that *X*_*ig*_ ∼ Poisson(*μ*_*ig*_) in [19].

Let *K* be the total number of classes and *C*_*k*_∈{1,…,*n*} the indices of samples in class *k* for *k*=1,…,*K*. Then the class-specific model for RNA-Seq data is given by

2 $$\begin{array}{@{}rcl@{}} (X_{ig}|y_{i}=k) ~\sim ~ \text{NB}(\mu_{ig}d_{kg},\phi_{g}), \end{array} $$

where *d*_*kg*_ are gene- and class-specific parameters that allow for differential expression among the K classes, and *y*_*i*_=*k*∈{1,…,*K*} represents the label of sample *i*. We also follow the independence assumption in PLDA [19] that all genes are independent of each other. Note that the independence assumption is frequently assumed in microarray data analysis. In real-world applications, the gene expression profile of an individual can be used to test whether this individual has a disease and/or a specific type of disease, which is essentially a classification problem.

Let $\mathbf {x}^{*}=(X_{1}^{*},\ldots,X_{G}^{*})^{T}$ be a test sample with *s*^∗^ the size factor and *y*^∗^ the class label. By Bayes’ rule, we have

3 $$\begin{array}{@{}rcl@{}} P(y^{*}=k|\mathbf{x}^{*}) ~\propto~ f_{k}(\mathbf{x}^{*})\pi_{k}, \end{array} $$

where *f*_*k*_ is the pdf of the sample in class *k*, and *π*_*k*_ is the prior probability that one sample comes from class *k*. The pdf of *X*_*ig*_=*x*_*ig*_ in model (2) is

4 $$\begin{array}{@{}rcl@{}} P(X_{ig}=x_{ig}|y_{i}=k) &=& \frac{\Gamma(x_{ig}+\phi_{g}^{-1})}{x_{ig}!\Gamma(\phi_{g}^{-1})} \left(\frac{s_{i} \lambda_{g} d_{kg} \phi_{g}}{1 + s_{i} \lambda_{g} d_{kg} \phi_{g}}\right)^{x_{ig}}  \\ &&\left(\frac{1}{1+s_{i}\lambda_{g}d_{kg}\phi_{g}}\right)^{\phi_{g}^{-1}}. \end{array} $$

By (3) and (4), we have the following discriminant score for NBLDA:

5 $$ {{}{\begin{aligned} \log P(y^{*}=k|\mathbf{x}^{*}) &=\sum\limits_{g=1}^{G} X_{g}^{*} \left[ \log d_{kg} - \log(1 + s^{*}\lambda_{g}d_{kg}\phi_{g})\right]\\ & \quad-\sum\limits_{g=1}^{G} \phi_{g}^{-1} \log(1+s^{*}\lambda_{g}d_{kg}\phi_{g}) + \log\pi_{k} + C, \end{aligned}}}  $$

where *C* is a constant independent of *k*. We then assign the new observation **x**^∗^ to class *k* that maximizes the quantity (5). Throughout the paper, we estimate the prior probability *π*_*k*_ by *n*_*k*_/*n*, where *n*_*k*_ is the sample size in class *k*. For balanced data, the prior probability is simplified as *π*_*k*_=1/*K* for all *k*=1,…,*K*. For gene *g*, the total number of reads is $\lambda _{g}=\sum _{i=1}^{n} X_{ig}$, and the class difference *d*_*kg*_ can be estimated by $(\sum _{i\in C_{k}}X_{ig} +1)/(\sum _{i\in C_{k}}\hat {s}_{i}\hat {\lambda }_{g} +1)$, which is the same posterior mean for $\hat {d}_{kg}$ in [19] assuming a Gamma prior distribution for this parameter. Estimation of the unknown parameters including *s*_*i*_ and *ϕ*_*g*_ will be discussed in Section “Parameter estimation”.

To explore the relationship between the proposed NBLDA and the PLDA, we assume that *s*^∗^*λ*_*g*_*d*_*kg*_ are bounded. When *ϕ*_*g*_→0, we have log(1+*s*^∗^*λ*_*g*_*d*_*kg*_*ϕ*_*g*_)→0 and $\phi _{g}^{-1} \log (1+s^{*}\lambda _{g}d_{kg}\phi _{g}) = \log (1+s^{*}\lambda _{g}d_{kg}\phi _{g})^{\phi _{g}^{-1}} \to s^{*}\lambda _{g}d_{kg}$.

Then consequently,

6 $$\begin{array}{@{}rcl@{}} \log P(y^{*}=k|\mathbf{x}^{*}) &\approx& \sum\limits_{g=1}^{G} X_{g}^{*} \log d_{kg} \\&-& \sum\limits_{g=1}^{G} s^{*}\lambda_{g}d_{kg} + \log\pi_{k} + C, \end{array} $$

where the right hand of (6) is the discriminant score of PLDA. That is, the NBLDA classifier reduces to the PLDA classifier when there is little dispersion in the data. From this point of view, the proposed NBLDA can be treated as a generalized version of PLDA.

To investigate how the dispersion changes their discriminant scores, We conduct a numerical comparison between NBLDA and PLDA. Two cases are considered, where the first one assumes a common dispersion for all genes, and the second one assumes not. Note that the classifiers (5) and (6) have two same terms: log*π*_*k*_ and *C*. Without loss of generality, we compute the discriminant scores only using the first two terms in (5) and (6), respectively. In the comparison study, we fix $X^{*}_{g}=10$, *d*_*kg*_=1.5, *s*^∗^=1, *λ*_*g*_=10 and *G*=500. For the case of common dispersion, we set the dispersion ranging from 0 to 20. For the case of different dispersions, we let *ϕ*_*g*_ be independent and identically distributed (i.i.d.) random variables from a chi-squared distribution with the degrees of freedom ranging from 0.1 to 5.

**Fig. 1:**
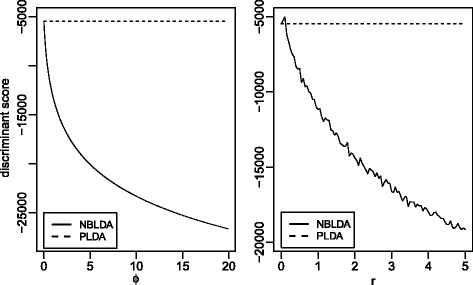
Numerical comparisons between NBLDA and PLDA. The left panel shows the results with a common dispersion *ϕ*. The right panel shows the results with different gene-specific dispersions *ϕ*_*g*_ which are i.i.d. random variables from a chi-squared distribution with *r* degrees of freedom. We compute the discriminant scores of NBLDA and PLDA for different *ϕ* and *r*

Figure 1 exhibits the comparison results. The left panel shows the results for the case of a common dispersion. Note that the discriminant score of PLDA is independent of the dispersion parameter and hence is a constant. For NBLDA, its discriminant score is a curve, and the slope is large for low dispersions and small for high dispersions. We find that the discriminant score of NBLDA is sensitive to the dispersion. Even when the dispersion is very small, the difference between the two discriminant scores is significant. The right panel in Fig. 1 shows the results for the case of different dispersions. The pattern of the right panel is similar to the left one except that the curve of NBLDA is not smooth. This suggests that, to analyze real data, we can first compute the median of the dispersions and then use such information to determine which classifier to use.

### Parameter estimation

Note that the discriminant score in (5) involves two unknown parameters, size factor *s*^∗^ and dispersion parameter *ϕ*_*g*_.

#### Size factor estimation

Due to different sequencing depths, the total number of reads differs across samples. Hence a normalization of the read counts through a size factor is a necessary step for analyzing RNA-Seq data [21, 22]. To estimate the size factor *s*_*i*_ for the training data and the size factor *s*^∗^ for the test data, we consider the following three procedures:

- *Total count*: PLDA divided the total read counts of sample *i* by the total read counts of all samples to estimate the size factor of sample *i*. That is,
  $$\hat{s}^{*}=\frac{\sum_{g=1}^{G} X^{*}_{g}}{\sum_{i=1}^{n}\sum_{g=1}^{G} X_{ig}}~~~\text{and}~~~\hat{s}_{i}=\frac{\sum_{g=1}^{G} X_{ig}}{\sum_{i=1}^{n}\sum_{g=1}^{G} X_{ig}}. $$
- *DESeq2*: Love et al. [11] first divided the read counts of sample *i* by the geometric mean of all samples’ read counts, and then estimated the size factor by computing the median of those *G* values. Specifically, the size factors are estimated by
  $$\begin{aligned} m^{*}&=\text{median}_{g}\frac{X_{g}^{*}}{\left(\prod_{i=1}^{n} X_{ig}\right)^{1/n}}~~~\text{and}\\&~~~m_{i}=\text{median}_{g}\frac{X_{ig}}{\left(\prod_{i=1}^{n} X_{ig}\right)^{1/n}}, \end{aligned} $$
  $$\hat{s}^{*}=m^{*}/\sum\limits^{n}_{i=1}m_{i}~~~\text{and}~~~\hat{s}_{i}=m_{i}/\sum\limits^{n}_{i=1}m_{i}. $$
- *Upper quartile*: Bullard et al. [21] proposed a robust method that uses the upper quartile of the read counts to estimate the size factors. Specifically, the size factors are estimated by
  $$\hat{s}^{*}=\frac{q^{*}}{\sum_{i=1}^{n} q_{i}}~~~\text{and}~~~\hat{s}_{i}=\frac{q_{i}}{\sum_{i=1}^{n} q_{i}}, $$
  where *q*^∗^ and *q*_*i*_ are the upper quartiles for the test data and sample *i* in the training data, respectively.

In our simulation studies, we find that there is little difference in the performance of classification among the three methods. Hence, for brevity, we only report the simulation results based on the total count method in the reminder of the paper.

#### Dispersion parameter estimation

Various methods for estimating the dispersion parameter *ϕ*_*g*_ have been proposed in the literature [9–12]. A comparative study [23] is also available where the authors investigated the influence of different dispersion parameter estimates on detecting differentially expressed genes in RNA-Seq data. More recently, Yu et al. [17] proposed a shrinkage estimator for *ϕ*_*g*_ that shrinks the gene-specific estimation towards a target value. Specifically, the dispersion estimator is estimated by

7 $$\begin{array}{@{}rcl@{}} \hat{\phi}_{g} = \delta\xi + (1-\delta)\tilde{\phi}_{g}, \end{array} $$

where *δ* is a weight defined as

$$\begin{array}{@{}rcl@{}} \delta = \frac{\sum_{g=1}^{G}\left\{\tilde{\phi}_{g}-(1/G)\sum_{g=1}^{G}\tilde{\phi}_{g}\right\}^{2}/(G-1)}{\sum_{g=1}^{G}\left(\tilde{\phi}_{g}-\xi\right)^{2}/(G-2)},  \end{array} $$

$\tilde {\phi }_{g}$ are the initial dispersion estimates obtained by the method of moments, and *ξ* is the target value calculated by minimizing the average squared difference between $\tilde {\phi }_{g}$ and $\hat {\phi }_{g}$. Throughout the paper, we use the estimator (7) to estimate the dispersion parameter.

## Results

In this section, we compare the performance of the following classification methods:

- NBLDA,
- PLDA,
- Support vector machines (SVM),
- K-nearest neighbors (KNN).

For PLDA, we use the R package “PoiClaClu". For SVM, we use the R package “e1071" and choose the radial basis kernel in our simulation studies. For KNN, we choose *k*=1, 3 and 5.

### Simulation design

We generate the data from the following negative binomial distribution:

8 $$\begin{array}{@{}rcl@{}} \left(X_{ig}|y_{i}=k\right) ~\sim~ \text{NB}(s_{i} \lambda_{g} d_{kg},\phi). \end{array} $$

**Fig. 2:**
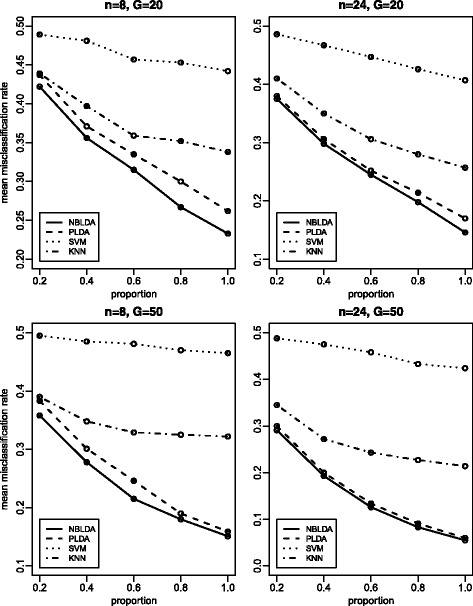
Mean misclassification rates for all four methods with *ϕ*=20 and *σ*=5. The x-axis represents the proportion of differentially expressed genes. 20, 40, 60, 80 and 100 % differentially expressed genes are considered, respectively. These plots investigate the effect of proportion of differentially expressed genes

**Fig. 3:**
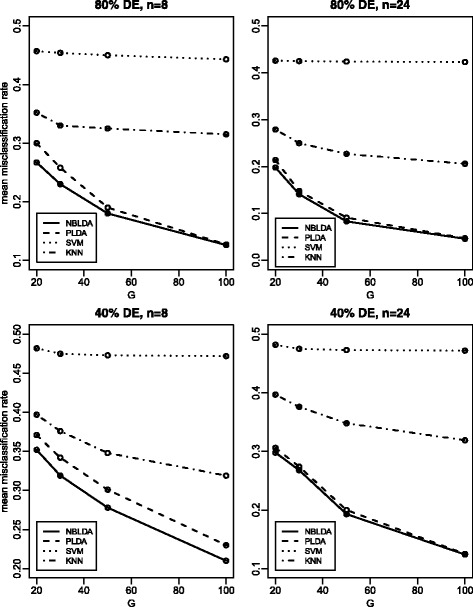
Mean misclassification rates for all four methods with *ϕ*=20 and *σ*=5. “80 % DE” means 80 % genes are differentially expressed, and the same to “40 % DE”. This plot investigates the effect of numbers of genes

**Fig. 4:**
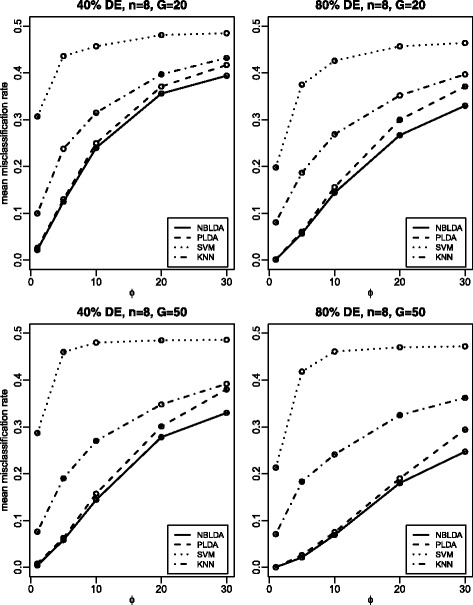
Mean misclassification rates for all four methods with *σ*=5. “80 % DE” means 80 % genes are differentially expressed, and the same to “40 % DE”. This plot investigates the effect of overdispersion

The total number of classes is *K*=2, and both the training data and test data have *n* samples. In all *G* genes, the proportions of differentially expressed genes are 0.2, 0.4, 0.6, 0.8 and 1.0, which represents that 20, 40, 60, 80 and 100 % genes are differentially expressed, respectively. For the differentially expressed genes, we set log*d*_*kg*_=*z*_*kg*_, where *z*_*kg*_ are i.i.d. random variables from the normal distribution *N*(0,*σ*^2^). For the constant genes, we set *d*_*kg*_=1. The size factors *s*_*i*_ are i.i.d. random variables from the uniform distribution on [0.2, 2.2]. The *λ*_*g*_ values are i.i.d. random variables from the exponential distribution with rate 0.04. Note that, for the sake of fairness, we have essentially followed the same simulation settings as those in PLDA. For the values of *G*, *n*, *ϕ* and *σ*, we specify them in Figs. 2, 3 and 4.

To compare these methods, we compute the mean misclassification rates as follows: for each simulation, we generate *n* test samples and compute the following misclassification rate:

$$\frac{\text{the~number~of~misclassified~samples}}{n}. $$

We run 1,000 simulations, compute its mean, and then obtain the mean misclassification rate. It is worth noting that Witten [19] discussed the problem of over-dispersion and proposed to transform the data to fit a Poisson model. In our experiment, we applied the data transformation proposed by Witten [19] when testing PLDA.

### Simulation results

Figure 2 illustrates the effect of the proportion of differentially expressed genes on the mean misclassification rate. In general, with an increasing number of differentially expressed genes, both methods have decreased mean classification rates. NBLDA always outperforms the other three methods. In particular, when the sample size is small (*n*=8), NBLDA has a significant improvement over the other approaches.

Figure 3 shows the impact of the number of genes on the mean misclassification rate. We consider *G*=20, 30, 50, and 100 for this investigation. From Fig. 3, we observe that an increasing number of genes will lead to a lower misclassification rate. NBLDA shows its superiority over the other three methods, and the improvement is more significant when the sample size and the number of genes are smaller.

Figure 4 shows the effect of overdispersion on the mean misclassification rate. We consider *ϕ*=1, 5, 10, 20 and 30 for this investigation. Figure 4 shows that a larger dispersion will result in a higher mean misclassification rate. Both NBLDA and PLDA perform better than SVM and KNN. When the overdispersion is not very high, NBLDA and PLDA have similar performance, with NBLDA slightly better than PLDA. When the overdispersion is high, however, the performance of NBLDA is much better than PLDA.

### Real data analysis

In this experiment, we use two real data sets to further compare our methods with the other methods. We note that SVM and KNN are applied on the log-transformed counts of these two real data sets. The reason is that in real data sets, the number of genes is large and their counts may exhibit largely different distributions. In this situation, a few strongly expressed genes with very large counts may dominate those weakly expressed genes, which may decrease the performance of SVM and KNN. However, in our simulation experiment, the number of genes is not big (100 maximum) and the counts of all expressed genes in one particular data set are from the negative binomial distribution with a common dispersion parameter. Such a situation has little chance to happen. Therefore, we directly applied SVM and KNN on the raw counts in the simulation experiment. The details of these two data sets are described as follows:

- *Cervical cancer data* [24]. Two groups of samples are contained in this data set. One is the nontumor group which includes 29 samples, and the other one is the tumor group which includes 29 samples. There are 714 microRNAs in this data set. This data set is available in Gene Expression Omnibus (GEO) Datasets with access number GSE20592.
- *HapMap data* [25, 26]. A total number of 52,580 probes are included in this data set, and this data set includes two classes, CEU and YRI, where the sample sizes are 60 and 69, respectively.

The Cervical cancer data was also used in [19]. It is worth mentioning that Witten [19] used four data sets to illustrate the performance of the proposed method. We found that for the other two data sets, i.e., Liver and kidney data and Yeast data, the mean misclassification rates of PLDA and NBLDA discussed in this paper are all zeros. The other data set, i.e., Transcription factor binding data, is the ChIP-Seq data. Hence, we do not discuss these three data sets in this manuscript.

#### Gene selection

For real biomedical research in which RNA-Seq technology is used, it is common that thousands or tens of thousands of genes are measured simultaneously. We perform a gene selection procedure to screen the informative genes before applying a classification rule to RNA-Seq data. By doing gene selection, we rule out the noise as much as possible so that the variance of the discriminant score is reduced, and consequently we have an increased interpretability.

The BSS/WSS method [27] is a common gene selection method and has been widely used in the literature [28–30]. This method computes the ratio of the sum of squares between groups to the sum of squares within groups for each gene, and selects genes whose ratios are in the top. However, this method assumes the data to be normally distributed so that it may not be suitable for RNA-Seq data.

Witten [19] proposed a screening method to select genes for RNA-Seq data by using soft-thresholding to shrink the estimate of *d*_*kg*_ towards 1. However, this method can not be applied to our discriminant analysis because the dispersion is involved in our discriminant rule. For the negative binomial distribution, edgeR [9, 10] has been proposed to detect differentially expressed genes in RNA-Seq data. This method first estimates the gene-wise dispersions by maximizing the combination of gene-specific conditional likelihood and common conditional likelihood, and then replaces the hypergeometric distribution in Fisher’s exact test by the negative binomial distribution to construct an exact test. In this paper, we use edgeR (version 3.3) to perform the gene selection procedure, which is available in Bioconductor (www.bioconductor.org).

#### Real data analysis results

We first conduct the gene selection procedure using edgeR (version 3.3) and obtain *G* genes for further analysis. We then randomly split the sample into two sets: the training set and the test set. The training set is used to construct the classifier and the test set is used to compute the misclassification rate. We repeat the whole procedure 1,000 times and compute the mean misclassification rate for the four methods, NBLDA, PLDA, SVM, and KNN, respectively.

**Fig. 5:**
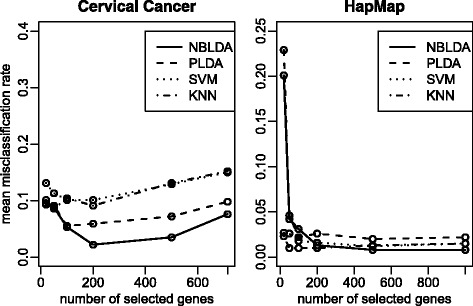
Mean misclassification rates for real data sets

The comparison results are shown in Fig. 5. For Cervical cancer data, 52 samples are assigned to the training set and 6 samples to the test set. A total of 20, 50, 100, 200, 500 and 714 genes are selected, respectively. Among all approaches we consider in this paper, our proposed NBLDA has the lowest misclassification rate. A big improvement over the other approaches can be observed when more than 50 genes are selected. For HapMap data, we randomly assign 70 samples to the training set and the remaining samples to the test set. A total of 20, 50, 100, 200, 500 and 1000 genes are selected, respectively. We can obtain similar results for HapMap data in Fig. 5.

Finally, we estimate the medians of the dispersions of these two data sets to check if it also supports our comparison results made in the previous paragraph. The simplest way for estimating the dispersion is to use the method of moments. However, this estimate may not be reliable (sometimes is a negative value) when the sample size is small. Landau and Liu [23] and Yu et al. [17] recently reviewed several dispersion estimation methods. For Cervical cancer data and HapMap data, we compute the medians of their dispersions using the method in Yu’s method [17] and present the estimates in Table 1. We note that these two data sets possess a considerably high dispersion when the number of selected genes is not very large. This, together with the numerical comparison in Fig. 1, explains why NBLDA provides a better performance than PLDA for these two data sets.

**Table 1:** The medians of their dispersions for Cervical cancer data and HapMap data, where "*G*" represents the number of top genes selected by edgeR (version 3.3)

Data sets	*G*=20	*G*=50	*G*=100	*G*=500
Cervical cancer	21.2	23.3	18.2	11.0
HapMap	36.4	40.1	38.2	20.1

## Discussion

In this paper, we have proposed an NBLDA classifier using the negative binomial model. Our simulation results show that our proposed NBLDA has a better performance than PLDA in the presence of moderate or high dispersions. When there is little dispersion in the data, NBLDA is also comparable to PLDA. We have further explored the relationship between NBLDA and PLDA, and investigated the impact of dispersion on the discriminant score of NBLDA by conducting a numerical comparison. It is worth noting that even for a small dispersion, the two discriminant scores can be rather different. This suggests that for real RNA-Seq data with moderate or high dispersion, NBLDA may be a more appropriate method than PLDA. Note that the true dispersions are unlikely to be known in practice. Therefore, we propose to first estimate the average dispersion using some novel estimation methods in the recent literature. Second, if the estimated average dispersion is small, we use PLDA; and otherwise we use NBLDA.

We note that the independence assumption in both Witten’s method and our method is very restrictive. For real gene expression data sets, it may not be realistic to assume that all genes are independent of each other. In our future study, we would like to incorporate the network information of pathways or gene sets to further improve the performance of classification. The clustering of sequencing data is also an important issue in biomedical research. Hence, another possible future work is to extend the proposed clustering method [19] to follow the negative binomial model.

## Conclusions

Next generation sequencing technology has been widely applied in biomedical research and RNA-Seq begins to replace the microarray technology gradually in recent years. Since RNA-Seq data are nonnegative integers, differing from that of microarray data, it is necessary to develop methods that are well suited for RNA-Seq data. Two discrete distributions, the Poisson distribution and negative binomial distribution, are commonly used in the literature to model RNA-Seq data. Compared to the Poisson distribution, the negative binomial distribution allows its variance to exceed its mean and is more suitable for the situations when biological replicates are available. Nevertheless, the negative binomial model is more complicated than the Poisson model as the additional dispersion parameter also needs to be estimated. In this paper, we have developed a new classifier using the negative binomial model for RNA-seq data classification. Our simulation results show that our proposed classifier has a better performance than existing works. To conclude, our proposed classifier can serve as an effective tool for classifying RNA-seq data.
